# PsiAudit: An open-source toolkit for auditing symmetry-organised complexity in equivariant quantum neural networks

**DOI:** 10.1371/journal.pone.0353739

**Published:** 2026-07-17

**Authors:** Hassan Ugail, Newton Howard

**Affiliations:** 1 Centre for Visual Computing and Intelligent Systems, University of Bradford, Bradford, United Kingdom; 2 School of Individualized Study, Rochester Institute of Technology, New York, New York, United States of America; Commonwealth Scientific and Industrial Research Organisation, AUSTRALIA

## Abstract

Parameterised quantum circuits are commonly assessed using measures such as expressibility, gradient behaviour, and entanglement. While useful, these measures do not indicate whether a circuit respects the symmetry it was designed to respect. This is especially important in equivariant quantum machine learning, where symmetry is central to the model. We introduce PsiAudit, an open-source Python toolkit for auditing symmetry-aware quantum neural network ansätze before training. Given an ansatz, a target symmetry, and a state trajectory, PsiAudit reports how the circuit occupies symmetry sectors, maintains coherence between them, fluctuates across the trajectory, and complies with the target symmetry. These outputs are combined into a configurable dashboard-style summary. PsiAudit supports phase, spin, and permutation symmetries, with the permutation audit implemented using Hamming-weight orbits. Tests on five ansatz families, across twenty random seeds and four to eight qubits, show, in the tested setting, that PsiAudit can identify inactive equivariant circuits, recover structure when multiple symmetry sectors are activated, and distinguish ansätze that appear similar under standard diagnostics. The toolkit also includes a unitary-level compliance check, reproducible examples, and a notebook for regenerating the reported results.

## 1. Introduction

Parameterised quantum circuits sit at the centre of quantum machine learning, variational quantum algorithms, and near-term quantum simulation [1–4]. In practice, before a candidate circuit is trained on hardware or on a high-fidelity simulator, a user will usually evaluate it with task-independent diagnostics. The reason is straightforward. The choice of ansatz affects trainability, resource requirements, inductive bias, and the range of states the model can reach [5–7]. In a near-term setting where shots, qubits, and depth are all limited, a diagnostic that flags undesirable behaviour before training is more valuable than one that requires a full training run to evaluate.

Several diagnostics are widely used. Expressibility quantifies how closely the distribution of states reachable by an ansatz resembles a Haar-random reference distribution over Hilbert space [8]. The variance of partial derivatives of a loss function is the standard probe for the barren-plateau pathology associated with deep parameterised circuits [9–11]. Meyer-Wallach *Q* summarises the average single-qubit entanglement of the output ensemble [12]. These quantities have been deployed extensively in studies of variational quantum classifiers, hybrid quantum-classical algorithms, and feature-map circuits [13–16]. They are useful and they are necessary, but each one of them is, by design, agnostic to the symmetry structure of the problem. None of them tells a practitioner whether the ansatz organises its trajectory in a way that respects a chosen target group.

That gap matters in equivariant quantum machine learning, where the symmetry group is part of the model design [17–22]. It dictates the allowed gate generators, the parameter-sharing pattern, the data-encoding map, and ultimately the inductive bias the architecture imposes. The same logic is well established in classical settings, where group-equivariant convolutional networks have shown that symmetry-aware models carry strong inductive biases when the data respect the group [23–25]. Two ansätze can share expressibility, gradient variance, and Meyer-Wallach *Q* while differing in how their trajectories populate symmetry sectors, fluctuate between them, and comply with the target group action at the level of their gate generators. A diagnostic toolkit for this setting therefore needs to report symmetry-organised complexity, not only generic complexity, while stating clearly what each component does and does not measure.

We present PsiAudit, an open-source Python toolkit that implements a preliminary, configurable audit framework for symmetry-organised complexity in equivariant quantum neural networks. The name reflects the two roles of the toolkit. The prefix refers to the quantum state vector ψ whose symmetry organisation is being measured, while “audit” denotes a pre-deployment diagnostic screen of ansatz behaviour rather than a trained-model evaluation. It supports three target groups, the unitary group *U*(1), the special unitary group *SU*(2), and the symmetric group $S_{n}$ implemented here through Hamming-weight orbit projectors. Given a parameterised ansatz, a target group, and a trajectory specification, it computes four normalised components, the sector-occupation entropy $H_{G}$, the cross-sector coherence proxy $D_{G}$, the sectoral fluctuation amplitude $M_{G}$, and the generator-sum compliance factor $S_{G}$, and combines them into a configurable composite index $\Psi_{G}$ that should be read as a dashboard summary of the four-component profile rather than as an invariant scalar measure of ansatz quality. The audit values are not predictions of classification accuracy, training cost, or hardware performance and should not be read as such. The toolkit ships with a reference ansatz library, implementations of the standard diagnostics for comparison, two trajectory builders, and a unit-test battery that validates the projector constructions and the boundedness of the components.

### 1.1. Scope and contributions

The contribution is principally a software toolkit that implements a preliminary, configurable audit framework whose mathematical primitives are derived from a companion theoretical study [26] and are stated here in self-contained form. The framework is preliminary in that the four components and their composite serve as practical proxies rather than fully validated invariants, and we say so throughout. Concretely, we provide a reproducible implementation of the four-component pipeline together with all sector-projector constructions, the generator-sum compliance defect, and a reference ansatz library spanning hardware-efficient [5], equivariant [17,18,20], and symmetry-broken constructions.

We audit five ansätze under both sector-confined and multi-sector trajectory regimes and compare $\Psi_{G}$ against expressibility [8], gradient variance [9], and Meyer-Wallach *Q* [12], identifying a regime where the standard diagnostics cluster equivariant and symmetry-broken ansätze together while the audit profile separates them through the fluctuation and compliance components, with a unitary-level check that sharpens the separation. Every reported quantity is replicated across 20 seeds and across n∈{4,6,8}, with confidence intervals. The induced ranking is shown to be robust to the composite weights and penalty through a sweep over 200 weight vectors and a grid of penalties. A downstream study on symmetry-matched tasks shows that task performance depends on the alignment between the task and the symmetry of the architecture. We explicitly state the implementation’s limitations, including the orbit decomposition used for $S_{n}$, the generator-sum nature of the compliance proxy, and the saturation of the multiplicity-space contribution to $D_{G}$.

The remainder of the paper is organised as follows. Section 2 describes the audit framework, the supported groups, the reference ansätze, the trajectory regimes, the comparative baselines, and the experimental design. Section 3 presents the empirical results. Section 4 interprets them and states the limitations, and Section 5 summarises the contribution. Appendix A gives the mathematical definitions, the sector-projector constructions, and the unit-test battery, and Appendix B documents the software API with a worked example.

## 2. Materials and methods

### 2.1. Ethics statement

This study did not involve human participants, animal experimentation, or the collection of personal data. All experiments used numerical simulations of parameterised quantum circuits implemented in publicly available open-source scientific Python libraries. No hardware experiments on human or animal subjects were performed. Therefore, no institutional ethics approval was required to undertake this study.

### 2.2. Overview of the audit framework

PsiAudit is a Python package and a reproducible Python notebook for symmetry-aware ansatz auditing. The package is built on the standard scientific Python stack [27–30] and runs end to end on a CPU in approximately one hour at the configuration used in this paper.

The audit pipeline takes as input a parameterised ansatz ${𝒜}_{\theta }$ that maps a parameter vector $\theta \in {\mathbb{R}}^{P}$ and an initial state $|\psi_{0}\rangle$ to an output state |ψ(θ)⟩, a target symmetry group *G* drawn from $\left\{U(1),SU(2),S_{n}\right\}$, a trajectory specification 𝒯 that produces an ordered ensemble of *T* states, a component-weight vector $𝐰=(w_{H},w_{D},w_{M})$ with $w_{H}+w_{D}+w_{M}=1$, and a compliance penalty parameter γ>0. The pipeline returns four normalised audit components on [0,1], namely the sector-occupation entropy $H_{G}$, the operational cross-sector coherence proxy $D_{G}$, the sectoral fluctuation amplitude $M_{G}$, and the generator-sum compliance factor $S_{G}$, together with the configurable composite audit score $\Psi_{G}$. It also writes a one-page audit report, raw CSV tables of the audit and comparative-diagnostic outputs, sector-occupation heatmaps, and a metadata JSON recording configuration, package versions, and random seeds.

### 2.3. The composite score and its components

We define the configurable composite audit index as the symmetry-compliance-weighted gated combination,


$$\begin{array}{c} \Psi_{G}\;=\;S_{G}\;(\;w_{H}\;H_{G}+w_{D}\;D_{G}+w_{M}\;H_{G}\;(1-M_{G})\;), \end{array}$$ (1)

in which $H_{G}$, $D_{G}$, and $M_{G}$ are derived from the state-trajectory ensemble produced by 𝒯 on ${𝒜}_{\theta }$, and $S_{G}$ is the generator-sum compliance factor derived from the ansatz operator structure. The first two terms reward sector spread and cross-sector coherence, respectively. The third term rewards stability $\left(1-M_{G}\right)$, but is gated by $H_{G}$ so that the stability contribution is active only when the sector structure itself is activated. Under the gated form, a sector-confined trajectory with $H_{G}=D_{G}=0$ collapses to $\Psi_{G}=0$ regardless of $M_{G}$ or $S_{G}$. Practitioners who care more about sector spread than about coherence, or who wish to remove the compliance penalty, can change the weights or set γ=0 without modifying the underlying audit components.

We use the conventional values $\left(w_{H},w_{D},w_{M})=(0.40,0.35,0.25\right)$ and γ=3 throughout the empirical study. These values give roughly equal influence to the three positive components when all three are non-trivial, while letting the compliance proxy attenuate the composite for non-equivariant generator-sum structures. The four-component profile and the composite are reported together so that the reader can inspect each axis directly. We do not claim that these weights are optimal in any task-specific sense. They are a convention.

The sector-occupation entropy measures how widely the trajectory occupies the symmetry sectors of *G*. For a target group with *K* sectors $\{\Lambda_{\lambda }{\}}_{\lambda =1}^{K}$ and an averaged trajectory occupation vector $\bar{p}=({\bar{p}}_{1},\ldots ,{\bar{p}}_{K})$, the normalised Shannon entropy is,


$$\begin{array}{c} H_{G}\;=\;-\;\frac{1}{\log K}\sum_{\lambda =1}^{K}{\bar{p}}_{\lambda }\log{\bar{p}}_{\lambda }. \end{array}$$ (2)

The normalisation places $H_{G}$ on [0, 1]. A trajectory that visits a single sector has $H_{G}=0$, while a trajectory that visits all sectors uniformly has $H_{G}=1$.

The cross-sector coherence proxy combines two contributions. The inter-sector Frobenius mass measures coherence between distinct symmetry sectors. For each state $|\psi_{t}\rangle$ in the trajectory, the inter-sector contribution is,


$$\begin{array}{c} D_{G}^{inter}(\psi_{t})\;=\;2\sum_{\lambda <\mu }{\left\|P_{\lambda }\;|\psi_{t}\rangle \langle \psi_{t}|\;P_{\mu }\right\|}_{F}, \end{array}$$ (3)

clipped to [0,1], where $\|\cdot {\|}_{F}$ denotes the Frobenius norm, and the factor of two implements the empirical normalisation adopted by the implementation. The intra-sector multiplicity-space contribution $D_{G}^{mult}$ measures off-diagonal coherence within a symmetry sector and its multiplicity copies, when the underlying representation has non-trivial multiplicity. For target groups whose physically relevant sectors have unit multiplicity in the analysed setting, $D_{G}^{mult}$ takes the empirical clip value. The combined cross-sector coherence proxy is,


$$\begin{array}{c} D_{G}\;=\;\frac{1}{2}\left[\;\overset{―}{D_{G}^{inter}}+\overset{―}{D_{G}^{mult}}\;\right], \end{array}$$ (4)

in which the overline denotes the mean over trajectory steps. We label this quantity an operational coherence proxy rather than a representation-theoretic coherence measure. The empirical normalisation in Equation (4) can saturate at the clip cap in sectors with non-trivial multiplicity, and we observe this saturation in our experiments at n≥6. As a result, the discriminative power of $D_{G}$ in the present implementation comes primarily from $D_{G}^{inter}$. A dimension-aware normalisation that scales by $\sqrt{d_{\lambda }(d_{\lambda }-1)}$ rather than by a fixed factor of one half would yield a non-saturating multiplicity contribution and is a natural next step for the package.

The sectoral fluctuation $M_{G}$ measures the step-to-step variability of sectoral occupation along the trajectory. For each step *t*, the inverse participation ra*t*io $R_{t}=\sum_{\lambda }p_{\lambda }(t{)}^{2}$ summarises how concentrated the sector occupation is at that step. The fluctuation component is,


$$\begin{array}{c} M_{G}\;=\;\min\;(1,\;2\;\sigma_{t}(R_{t})), \end{array}$$ (5)

where $\sigma_{t}$ denotes the standard deviation over trajectory steps. Larger $M_{G}$ values indicate greater step-to-step variability in how concentrated the sectoral occupation is, which is associated with symmetry-broken dynamics that allow the trajectory to fluctuate across the sector ladder. Equivariant ansatz dynamics in regimes where the ansatz preserves the relevant sectors tend to produce small $M_{G}$. The composite in Equation (1) uses the complementary quantity $1-M_{G}$, which we interpret as a stability contribution.

The compliance factor measures how closely the gate-generator structure of the ansatz respects the target group action. We use a generator-sum formulation. Let $\{G_{a}{\}}_{a=1}^{n_{g}}$ be a generator set of the Lie algebra of *G*, or in the discrete-group case, a complete set of group generators. Given an effective ansatz Hamiltonian $H_{𝒜}$ defined as the sum of the gate generators in the parameterised circuit, the normalised commutator defect is,


$$\begin{array}{c} \Delta_{G}\;=\;\frac{1}{n_{g}}\sum_{a=1}^{n_{g}}\frac{{\left\|H_{𝒜}\;G_{a}-G_{a}\;H_{𝒜}\right\|}_{F}}{{\left\|H_{𝒜}\right\|}_{F}\;{\left\|G_{a}\right\|}_{F}}, \end{array}$$ (6)

and the generator-sum compliance factor is then $S_{G}=\exp(-\gamma \;\Delta_{G})$. We emphasise that this is a generator-sum proxy rather than a circuit-level equivariance check. A generator sum can look symmetric even when individual circuit instances do not commute with the target symmetry, for example through gate ordering or fixed non-infinitesimal local rotations that cancel under the linear sum but not under the unitary product. The score $S_{G}$ should therefore be read as a fast structural proxy. To complement it, PsiAudit also reports a unitary-level compliance check for the natural-group cases as a standard part of the audit output. This second diagnostic is more expensive because it samples realised circuit unitaries across a parameter ensemble, but it directly tests whether the implemented circuit commutes with the target action rather than relying on the generator sum; therefore, it is the more stringent of the two compliance measures. The channel-level version required for noisy circuits described by quantum channels is developed in a related study [31].

### 2.4. Supported target groups, the $S_{n}$ hamming-weight orbit audit, and reference ansätze

PsiAudit currently supports three target groups, chosen to span Abelian, non-Abelian Lie, and finite-discrete symmetries. The Abelian group *U*(1) generates phase rotations and admits a particle-number-preserving sector decomposition on *n* qubits. The non-Abelian Lie group *SU*(2) generates global spin rotations and admits a total-spin sector decomposition through Schur-Weyl duality [32]. The symmetric group $S_{n}$ generates permutations of the qubits. For *U*(1), the package implements particle-number projectors with sector labels k∈{0,1,…,n}. For *SU*(2), the package implements total-spin sector projectors with sector labels $j\in \{0,\frac{1}{2},\ldots ,\frac{n}{2}\}$ assembled from the Schur-Weyl decomposition. For $S_{n}$, the package implements a Hamming-weight orbit decomposition. We describe the audit against $S_{n}$ throughout as an $S_{n}$ Hamming-weight orbit audit. The orbit decomposition correctly identifies the trivial-irrep occupation pattern and is sufficient for the audit primitives implemented here, but it does not separate the multiplicity space of the standard (n−1,1) irrep, the sign irrep, or higher Young-diagram irreps [22,33]. The full implementation details for all three groups are stated in Appendix A.

To evaluate the audit toolkit, we provide five reference ansatz families that span hardware-efficient, equivariant, and symmetry-broken constructions. The hardware-efficient family is a hardware-efficient ansatz of the kind introduced for variational quantum eigensolvers [5]. It uses arbitrary single-qubit rotations interleaved with a fixed two-qubit entangling pattern, has no symmetry constraint, and serves as the generic baseline. The *U*(1)-equivariant family uses a particle-number-preserving gate set consisting of Givens-style hopping rotations. Its generator sum commutes with the number operator, so the family is *U*(1)-equivariant at the generator-sum level [17,20]. The *SU*(2)-equivariant family uses Heisenberg-style two-qubit interaction generators together with global spin rotations. Its generator sum commutes with the total-spin generators $S_{x},S_{y},S_{z}$, so the family is *SU*(2)-equivariant at the generator-sum level [18,19]. The $S_{n}$-equivariant family follows a parameter-input form whose generators commute with all qubit permutations [18,22]. The *U*(1)-broken family takes the *U*(1)-equivariant base and appends a fixed local-X rotation on each qubit at the end of every layer. This is a *U*(1)-symmetry-breaking inserted at the gate level. It serves as the near-control against which the *U*(1)-equivariant ansatz is compared.

### 2.5. Trajectory regimes and comparative diagnostics

The audit framework supports two complementary trajectory regimes that exercise different aspects of the sector structure. In the sector-confined regime, which we refer to as Regime A, the trajectory starts from the computational-basis vacuum $|0\rangle^{⊗n}$. This state is supported entirely in the *k* = 0 sector for *U*(1) and in the *j* = *n*/2 sector for *SU*(2). Under any ansatz in which the generators all commute with the relevant target generators, the trajectory remains confined to that sector. This regime is faithful to the typical operational scenario in variational quantum algorithms, where the input state is a single computational-basis reference. In the multi-sector regime, which we refer to as Regime B, we use a product-state initialisation generated by independent single-qubit *y*-axis rotations applied to the vacuum. At trajectory step *t*, the initial sta*t*e is,


$$\begin{array}{c} |\psi_{0}(t)\rangle \;=\;\overset{n-1}{\underset{q=0}{⨂}}R_{y}\;(\alpha_{q}(t))\;|0\rangle^{⊗n},\;\alpha_{q}(t)\;=\;\frac{\pi }{2}\;(\;0.5+0.4\;\sin(t+2\pi q/n)\;). \end{array}$$ (7)

We use a product-state initialisation rather than a symmetric Dicke superposition. A product state with independent rotation angles has non-zero overlap with multiple computational-basis strings and therefore with multiple *U*(1) sectors. The same product state is not, in general, supported within a single *SU*(2) total-spin sector, so it activates more than one *j* when audited against *SU*(2). The angles in Equation (7) are bounded between approximately 0.16 and 1.41 radians, with a typical value near π/4, which gives each qubit an excitation probability of roughly 0.15. For *n* = 6 this places the expected occupation peak in the *k* = 1 *U*(1) sector rather than at the binomial *k* = *n*/2 peak that would be obtained from a fully symmetric Dicke superposition. We chose this initialisation because it is the simplest product-state construction that activates multiple sectors across all three target groups, as required by the audit components. A more symmetry-faithful initialisation that uses a properly normalised symmetric Dicke superposition is straightforward to add as an alternative trajectory builder. The expected occupation patterns under Equation (7) are described and validated in Appendix A.

Three standard diagnostics serve as the comparative baselines against $\Psi_{G}$. Expressibility is computed as the symmetric Kullback-Leibler divergence between the empirical fidelity distribution of the ansatz and the Haar fidelity distribution on the relevant Hilbert space, following the protocol of [8]. We use 200 random parameter samples per ansatz and 50 fidelity-distribution bins. Gradient variance is computed as the variance of $\partial \langle Z_{0}\rangle /\partial \theta_{0}$ under random initialisation [9,11], with 80 finite-difference random initialisations under central finite difference with $\epsilon =10^{-4}$. Meyer-Wallach *Q* is the average single-qubit linear-entropy entanglement of the output state averaged over 200 random parameter samples [12]. These three diagnostics provide the standard comparison surface against which $\Psi_{G}$ and its components are read.

### 2.6. Experimental design and statistical analysis

The audit grid at *n* = 6 uses circuit depth *L* = 2 and trajectory length *T* = 40, with both Regime A and Regime B evaluated for each of the five reference ansätze against all three target groups. The scaling experiment uses n∈{4,6,8} at the same depth and trajectory length in Regime B against the natural target group of each ansatz. The comparative diagnostics are computed at *n* = 6 from 200 random parameter samples for expressibility and Meyer-Wallach *Q* and from 80 finite-difference initialisations for gradient variance.

The primary observational unit is one ansatz-by-target-group-by-regime combination at a fixed (*n*, *L*, *T*) triple, for which the pipeline reports $H_{G}$, $D_{G}$, $M_{G}$, $S_{G}$, and $\Psi_{G}$ along with the three comparative diagnostics and the unitary-level commutator deviation, as described below. To characterise variability rather than report a single realisation, every quantity in the study is replicated across twenty independent random seeds that control ansatz initialisation, trajectory sampling, and the random-parameter draws used by the diagnostics. We report the mean across seeds, along with its standard deviation and a 95% confidence interval obtained by bootstrap resampling over the seeds. A single audit run is deterministic once its seed is fixed, so each replicate is reproducible exactly on the same hardware, and the spread across replicates reflects genuine seed-to-seed variation rather than numerical noise. The unitary-level commutator deviation is a structural property of the circuit that does not vary across seeds, so it is evaluated once and reported with the rest of the audit. No reported audit quantity therefore rests on a single realisation. The structural correctness of the underlying primitives is checked independently of these experiments by a unit-test battery. These tests verify the idempotence and completeness of the sector projectors, the vanishing of the compliance defect for the equivariant constructions against their target generators, the calibration of the entanglement diagnostic on reference states, and the normalisation bounds of the components. The full test battery passes at every system size used in this study.

The scope of inference is the five reference ansätze, three target groups, two regimes, system sizes up to *n* = 8, and the configuration parameters listed above. Cross-family comparisons among the reference ansätze are read descriptively rather than as estimates for the broader population of equivariant constructions. Beyond the multi-seed replication, we probe two further questions that bear directly on how the composite should be trusted. We test how sensitive the ranking of the five ansätze is to the composite weights and the compliance penalty by recomputing $\Psi_{G}$ over two hundred randomly drawn weight vectors and a grid of penalty values, measuring rank agreement against the convention setting through Kendall’s τ. We also measure the wall-clock cost of one audit as a function of qubit count up to *n* = 10, which characterises the computational reach of the method. The audit components are evaluated without forming the dense $2^{n}\times 2^{n}$ density matrix or circuit unitary. For pure states, the Frobenius-norm quantities required by the components can be obtained from projector and gate actions on the state vector, which keeps the audit tractable across the range studied here.

The downstream compatibility study uses its own training protocol, which is kept simple so that the result reflects structural compatibility rather than optimiser tuning. Each task is a balanced binary problem with 12 examples per class at *n* = 6, classified using the normalised total-*Z* expectation with a logistic loss. Each ansatz is trained on each task using simultaneous-perturbation stochastic approximation, which estimates the gradient from two loss evaluations per step regardless of the parameter count, and is run for 60 update steps at a learning rate of 0.3 with a perturbation size of 0.05. To characterise variability, every ansatz-and-task cell is trained from eight independent random initialisations, and we report the mean accuracy, loss, and convergence rate, along with their standard deviations, across these eight runs. The convergence rate is the fraction of the total loss reduction achieved within the first 10 steps, so a higher value indicates faster early learning. These training seeds are independent of the twenty seeds used for the audit replication, since the two studies probe different objects, the audit profile in one case and the trained model behaviour in the other.

## 3. Results

The empirical analysis proceeds through a sequence of results, each tied to a figure or a table. We begin by examining the sector-occupation trajectories produced by the audit pipeline. We then compare $\Psi_{G}$ against the three standard diagnostics on the same five ansätze and identify a clustering regime in which the standard diagnostics fail to separate ansätze that the audit components do separate. Next, we examine how the audit components behave across system sizes from four to eight qubits, reporting means and confidence intervals across twenty seeds at each size. We then quantify the contrast between Regime A and Regime B and document the exact sector-confinement collapse of equivariant ansätze under the gated composite, and we use a gate ablation to show that the collapse is a direct consequence of the way the stability term is gated. We report the full per-component audit profile at *n* = 6 in Regime B, including its seed-to-seed spread, and examine the robustness of the resulting ranking to the composite weights and the compliance penalty. Finally, we report a downstream compatibility study that relates the audit to performance on symmetry-matched classification tasks, and we summarise the computational cost of the audit as a function of system size. The gated composite of Equation (1) is used throughout.

### 3.1. Sector-occupation trajectories reveal architecture-specific structure

**Fig 1:**
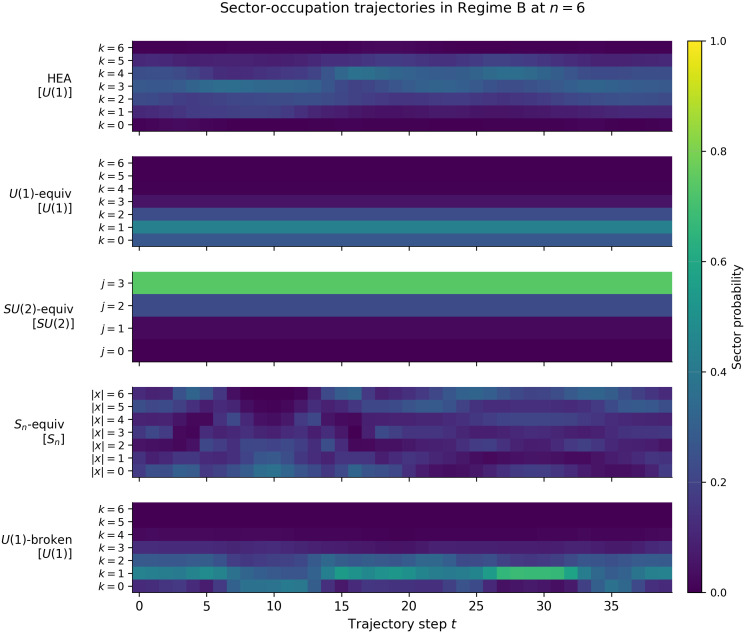
Sector-occupation trajectories at *n* = 6 in Regime B. Each row shows the sector-probability trajectory of one reference ansatz, audited against its natural target group. Rows from top to bottom show the hardware-efficient ansatz against *U*(1), the *U*(1)-equivariant ansatz against *U*(1), the *SU*(2)-equivariant ansatz against *SU*(2), the $S_{n}$-equivariant ansatz against the $S_{n}$ Hamming-weight orbit decomposition, and the *U*(1)-broken ansatz against *U*(1). Within each row, columns index trajectory steps and rows index symmetry sectors. The colour scale shows sector probabilities from 0 to 1. Equivariant ansätze produce stable sectoral occupation patterns inherited from the product-state initialisation in Equation (7), while the *U*(1)-broken ansatz develops a visibly drifting concentration.

Fig 1 shows the sector-occupation heatmaps that the pipeline records for each ansatz at *n* = 6 in Regime B. The hardware-efficient ansatz mixes broadly across all *U*(1) sectors with occupation that drifts slowly and never concentrates. The *U*(1)-equivariant ansatz keeps its occupation near the low-*k* end of the ladder, reflecting the product-state initialisation of Equation (7) whose typical angles give each qubit an excitation probability near 0.15 and place the peak in the *k* = 1 sector, and because its generators commute with the number operator, it preserves this pattern across the trajectory. The *SU*(2)-equivariant ansatz, audited against *SU*(2), is strongly stationary and dominated by the highest-spin *j* = 3 sector with residual support in *j* = 2, the latter because the product-state input is not fully contained in *j* = 3 and the ansatz preserves whatever spin content it is given. The $S_{n}$-equivariant ansatz occupies a broad range of Hamming-weight orbits with visible step-to-step variation, reflecting the richer orbit ladder open to a permutation-equivariant construction, while the *U*(1)-broken ansatz develops a pronounced and shifting concentration near *k* = 1 that is the signature of an explicit symmetry violation which nonetheless preserves much of the average occupation structure.

These panels motivate the use of the composite index. A statistic that summarised only sectoral spread would call the hardware-efficient and $S_{n}$-equivariant ansätze similar, while one that tracked only stability would call the *U*(1)- and *SU*(2)-equivariant ansätze similar. The pipeline records all four behaviours in a single pass and combines them, weighted by generator-sum compliance, into the configurable composite $\Psi_{G}$ in Equation (1).

### 3.2. The composite score separates architectures that standard diagnostics cluster together

**Fig 2:**
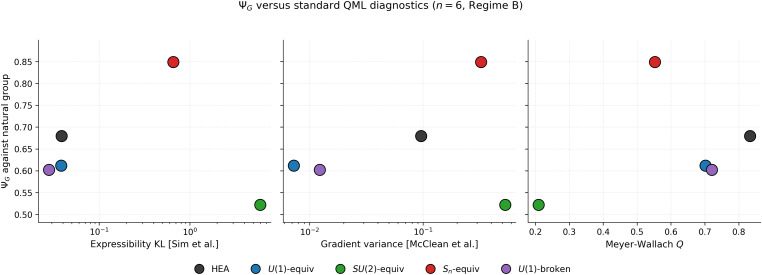
Corrected $\Psi_{G}$ versus standard quantum machine learning diagnostics at *n* = 6 in Regime B. Each panel plots the corrected $\Psi_{G}$ of Equation (1) audited against the natural target group of each ansatz on the vertical axis. The horizontal axes show the expressibility KL of [8] on the left, the gradient variance of [9] in the middle, and the Meyer-Wallach *Q* of [12] on the right. The five reference ansätze are coloured consistently across panels. Under each standard diagnostic, the *U*(1)-equivariant and *U*(1)-broken ansätze cluster closely together. Under the PsiAudit component profile, they separate, primarily through the sectoral fluctuation $M_{G}$ rather than through the composite alone.

Fig 2 compares the gated $\Psi_{G}$ against the three standard diagnostics for the five ansätze at *n* = 6 in Regime B, with $\Psi_{G}$ on the vertical axis and the expressibility KL, gradient variance, and Meyer-Wallach *Q* in turn on the horizontal axis. Two findings stand out.

The first concerns the *U*(1)-equivariant and *U*(1)-broken ansätze, which share a base structure and differ only by a fixed local-X rotation appended to each layer in the broken variant. Under every standard diagnostic they sit close together, with expressibility KL values of 0.038 and 0.028, gradient variances of 0.007 and 0.012, and Meyer-Wallach *Q* values of 0.702 and 0.721, so by any one of them the two look almost interchangeable. The audit separates them. Their composites are close, 0.612 against 0.624 as means over twenty seeds, but the components differ cleanly. The equivariant ansatz is exactly deterministic with $M_{G}=0$ and $S_{G}=1$, whereas the broken ansatz carries $M_{G}=0.067$ and a compliance that drops to $S_{G}=0.972$. The unitary-level commutator deviation of Equation (8) gives the sharpest reading, scoring 0 exactly for the equivariant ansatz and 0.027 for the broken one, with corresponding unitary-level compliance $S_{U}=0.922$. The circuit-level diagnostic and the trajectory-level fluctuation both expose the local-X breaking that the generator-sum gap of 0.028 is, by itself, too small to flag with confidence.

The second concerns the $S_{n}$-equivariant ansatz, which sits at intermediate values under all three standard diagnostics, with expressibility KL 0.656 between the hardware-efficient 0.038 and the *SU*(2)-equivariant 5.980, gradient variance 0.325 below the *SU*(2)-equivariant 0.530 and above the *U*(1)-family range of 0.007 to 0.096, and Meyer-Wallach *Q* of 0.553. Audited against its natural $S_{n}$ orbit target it attains the highest composite of the five, with a mean of 0.744 over twenty seeds, and it is the only one with appreciable seed variation, a standard deviation of 0.072 and a 95% confidence interval from 0.713 to 0.774, where the others are deterministic or vary by less than 0.02. That spread reflects the richer orbit-ladder permutation equivariance that opens up on the product-state input. The high mean reflects broad orbit occupation and exact compliance with the natural target in the multi-sector regime, and is conditional on that target and regime rather than a statement of general superiority, a point we return to in the compatibility study below.

### 3.3. Audit profiles are stable across seeds and system sizes

**Fig 3:**
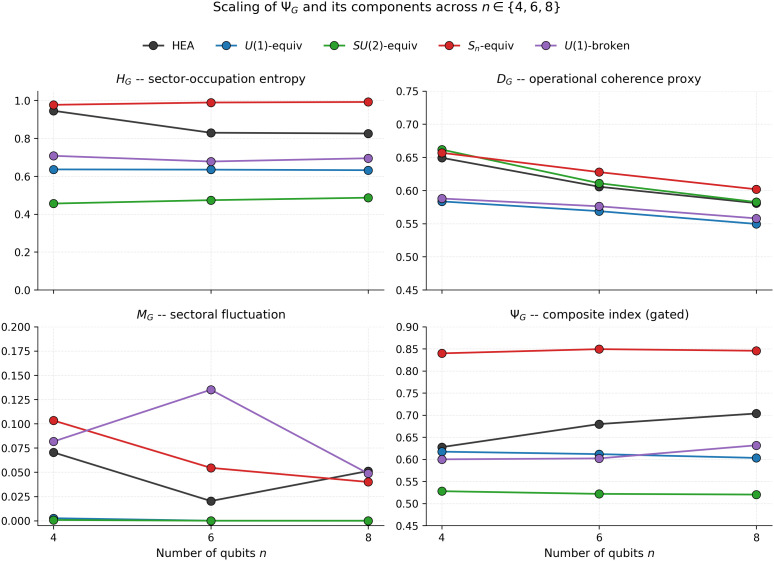
Scaling of the gated $\Psi_{G}$ and its components across n∈{4,6,8}. Each sub-panel shows one audit component on the vertical axis as a function of qubit count on the horizontal axis, with every point a mean over twenty seeds at fixed circuit depth *L* = 2 and trajectory length *T* = 40. Colours encode the five reference ansätze. The qualitative ordering of the ansätze on each component is preserved across the tested range of system sizes, and the seed-to-seed spread is small for every ansatz except the $S_{n}$-equivariant family. Values are computed using the gated composite of Equation (1).

Fig 3 shows the four components and the gated $\Psi_{G}$ against system size in Regime B; each point is the mean over twenty seeds. The ordering of the ansätze on every component is preserved across the tested range. The $S_{n}$-equivariant ansatz holds the highest $H_{G}$ at every size, followed by the hardware-efficient, *U*(1)-broken, *U*(1)-equivariant, and *SU*(2)-equivariant ansätze, while $D_{G}$ falls slowly for all of them as the enlarging sector list dilutes inter-sector coherence and $M_{G}$ stays small for the equivariant ansätze and larger for the *U*(1)-broken near-control. The composites are stable across both seeds and sizes. The *SU*(2)- and *U*(1)-equivariant ansätze are exactly deterministic at 0.520 to 0.528 and 0.603 to 0.612 respectively, the *U*(1)-broken composite moves from 0.599 at *n* = 4 to 0.629 at *n* = 8 with its seed spread shrinking from 0.019 to 0.012, the hardware-efficient ansatz shows the largest size dependence as it rises from 0.587 to 0.687 with growing compliance, and the $S_{n}$-equivariant composite stays near 0.74 while carrying the widest seed band at a standard deviation near 0.07.

Because each point aggregates 20 seeds, this stability reflects both sampling variation and system size. The narrow confidence bands on four of the five ansätze and the exact determinism of the two fully equivariant families indicate that the audit profile at *n* = 6 is representative of *n* = 4 and *n* = 8 under the same configuration, so a small-system audit can serve as a useful preliminary screen for deployment at slightly larger sizes in the same setting. We do not sweep trajectory length or depth, so we read this as a finite-size stability property at the studied depth rather than a claim of large-*n* universality. The wider band on the $S_{n}$-equivariant composite is itself informative, since it singles out the one architecture whose audit is genuinely sensitive to the random trajectory and therefore the one for which a confidence interval matters most.

### 3.4. Sector-confinement collapses equivariant ansätze to zero in regime A

**Fig 4:**
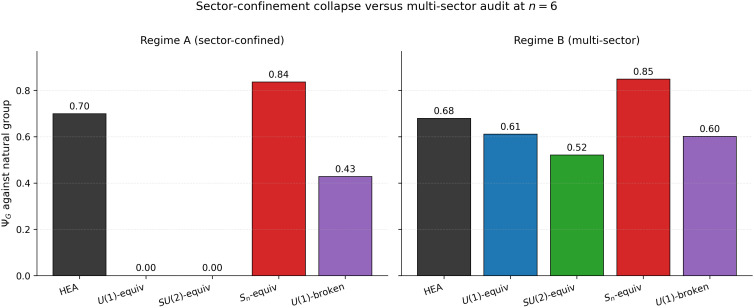
Sector-confinement collapse versus multi-sector audit at *n* = 6. Bars show the gated composite $\Psi_{G}$ of Equation (1) audited against the natural target group of each ansatz. The left panel shows Regime A, in which the trajectory starts from the computational-basis vacuum and equivariant ansätze remain sector-confined. The right panel shows Regime B, in which the trajectory starts from a product-state initialisation that spans multiple sectors. The *U*(1)-equivariant and *SU*(2)-equivariant ansätze collapse to $\Psi_{G}=0$ in Regime A, while the $S_{n}$-equivariant and *U*(1)-broken ansätze sit above zero in Regime A because their gate structure spreads the vacuum across multiple sectors or orbits. In Regime B, all five composites lie between approximately 0.52 and 0.85.

Fig 4 contrasts the two regimes, showing the composite audited against each ansatz’s natural group for the sector-confined trajectories starting from $|0\rangle^{⊗n}$ on the left and the multi-sector trajectories of Equation (7) on the right. In Regime A the *U*(1)- and *SU*(2)-equivariant ansätze collapse to $\Psi_{G}=0$ exactly. The vacuum lies in the *k* = 0 sector for *U*(1) and the *j* = 3 sector for *SU*(2), so any ansatz whose generators commute with the target generators stays confined there, the entropy and coherence components vanish, the $H_{G}$ gate on the stability term forces the third component to vanish too, and the composite is identically zero. The $S_{n}$-equivariant and *U*(1)-broken ansätze sit well above zero in Regime A, the former near 0.84 because its gate set does not preserve Hamming weight and spreads the vacuum across orbits, the latter near 0.43 because the deliberate breaking lets the trajectory leave the *k* = 0 sector. In Regime B, all five composites lie in a band between roughly 0.52 and 0.85.

The composite is therefore conditional on the input regime and is not an intrinsic architectural quantity. An equivariant ansatz that collapses to zero in Regime A is not defective. It is acting correctly under a degenerate input that does not exercise its symmetry structure, so a useful audit requires a regime that activates the relevant sectors, in keeping with the role of initialisation choices noted in the trainability and equivariant-learning literature [13–16,34]. The collapse is a designed property rather than an accident of the inputs, which a small ablation confirms. If the stability term is computed without the $H_{G}$ gate, so that it contributes through $1-M_{G}$ alone, the two confined equivariant ansätze no longer collapse but instead pick up a spurious floor of 0.250 in Regime A purely from the stability term acting on a degenerate trajectory. Gating that term by $H_{G}$ removes the artefact and reports that a confined trajectory carries no activated structure, so the gate is not a free parameter but the mechanism that gives the Regime A collapse its clean interpretation.

### 3.5. Component-level profile and robustness of the ranking

**Fig 5:**
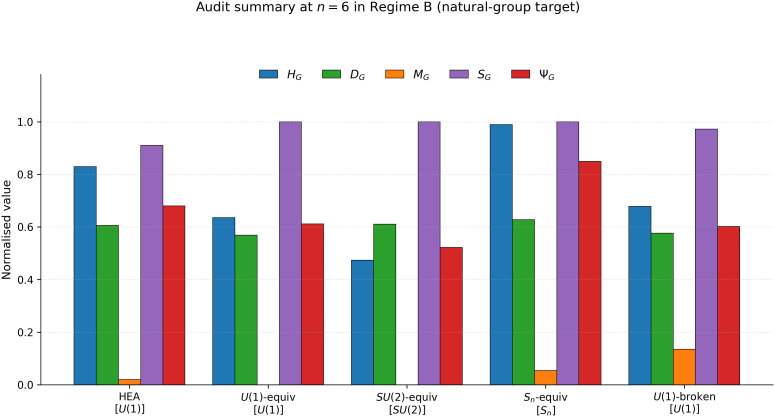
Component-level audit summary at *n* = 6 in Regime B. For each of the five reference ansätze, the bars show sector-occupation entropy $H_{G}$, the operational cross-sector coherence proxy $D_{G}$, the sectoral fluctuation $M_{G}$, the generator-sum compliance $S_{G}$, and the corrected composite audit index $\Psi_{G}$ of Equation (1), evaluated against the natural target group of each ansatz. The target group is shown in brackets below the ansatz label. $H_{G}$ and $S_{G}$ separate the ansätze on different axes. $M_{G}$ is small for the equivariant ansätze and visibly larger for the symmetry-broken near-control.

Fig 5 reports the full per-component summary at *n* = 6 in Regime B against the natural target groups, as means over twenty seeds. The hardware-efficient ansatz combines a high $H_{G}$ near 0.82, a moderate $D_{G}$ near 0.60, a near-zero $M_{G}$ of about 0.04, and compliance near 0.91 for a composite of 0.67. The *U*(1)-equivariant ansatz has $H_{G}\approx 0.63$, zero $M_{G}$, exact compliance, and a deterministic composite of 0.61, while the *SU*(2)-equivariant ansatz has a smaller $H_{G}$ of 0.47 from its concentration on the highest-spin sector, zero $M_{G}$, exact compliance, and a deterministic composite of 0.52. The $S_{n}$-equivariant ansatz has the highest $H_{G}$ near 0.88 and the highest composite near 0.74, with exact orbit-level compliance, and is the only profile with a wide seed band. The *U*(1)-broken ansatz has $H_{G}\approx 0.69$, a visibly larger $M_{G}$ near 0.07, and a near-compliant $S_{G}$ of 0.97 that is small enough that the generator-sum proxy alone would not flag it with confidence, which is exactly what the unitary-level deviation of Equation (8) is for. The profile makes the design point concrete. The composite compresses a four-dimensional reading into one number, so a reader screening equivariant from non-equivariant constructions can consult $S_{G}$ but should not rely on it alone. A reader interested in distinguishing confined from multi-sector trajectories can consult $H_{G}$, while a reader interested in the persistence of occupation can consult $M_{G}$.

Because the composite combines the four components through a weight vector and a penalty, it is fair to ask whether the ranking it induces is an artefact of those choices. Recomputing $\Psi_{G}$ over two hundred weight vectors drawn from a Dirichlet distribution over the three positive components and over a grid of penalties γ∈{0,1,2,3,5,8}, and comparing each ranking against the convention setting through Kendall’s τ, the ordering proves highly stable. The median rank agreement across the weight draws is τ=1.0 with a mean of 0.965, and the highest-scoring ansatz is unchanged in every draw, with reordering confined to adjacent lower-ranked ansätze under extreme weight vectors. Across the whole penalty grid the agreement is exact in this experiment, with τ=1.0 at every tested value of γ. This reflects the fact that the five ansätze are well separated on the components the penalty acts through, so changing γ does not reorder them here, rather than a general guarantee that the penalty preserves order, since ansätze with different compliance defects could in principle reorder under a different penalty. The four-component profile remains the primary object, but the ordering produced by the composite does not depend on the particular weights and penalty adopted here.

### 3.6. Downstream compatibility is governed by symmetry structure

A recurring question for any diagnostic is whether it relates to task performance, and answering it for equivariant models needs care, since an ansatz equivariant to a symmetry cannot distinguish data differing only along that symmetry’s quotient, so no single fixed task is equally fair to all five architectures. The study that follows is not intended as a benchmark of classifier performance, but as a controlled check that the audit profile reflects the symmetry compatibility between the ansatz and the task. Rather than tune a single task, we defined three fixed binary tasks matched to the three target symmetries. These were a *U*(1) task separating states of different total excitation, an *SU*(2) task separating collective-spin configurations, and an $S_{n}$ task separating permutation-symmetric states of different collective angle. All three tasks were read out via the total magnetisation, and each ansatz was trained on each task using simultaneous-perturbation gradient descent. The tasks and readout are fixed in advance and not adjusted per ansatz, so the resulting accuracy matrix is a structural result rather than a constructed one.

The matrix shows that compatibility, not raw capacity, decides which ansatz learns which task. Each accuracy is a mean over eight training runs, with zero spread for the clearly incompatible pairs and the deterministic equivariant cases and at most 0.10 elsewhere, so the pattern is stable rather than an optimisation artefact. The unconstrained hardware-efficient ansatz learns all three tasks, reaching perfect accuracy on the *SU*(2) and $S_{n}$ tasks and 0.83 on the *U*(1) task. The $S_{n}$-equivariant ansatz solves the $S_{n}$ and *U*(1) tasks perfectly, since permutation equivariance subsumes the excitation structure that the *U*(1) task depends on, and reaches 0.67 on the *SU*(2) task. The *U*(1)-equivariant, *SU*(2)-equivariant, and *U*(1)-broken ansätze succeed only on the *SU*(2) task and sit at chance on the others, because the discriminating feature of those tasks lies where their structure either preserves or averages it away under a total-magnetisation readout. Downstream performance for an equivariant model is therefore irreducibly relative to the task, and an architecture is useful precisely on problems whose structure aligns with its symmetry.

**Fig 6:**
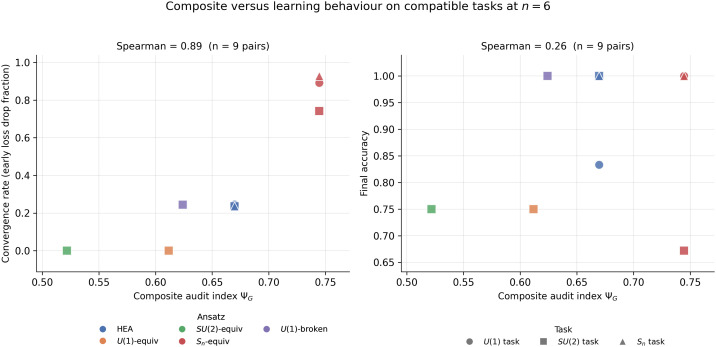
Composite versus learning behaviour on compatible tasks at *n* = 6. Each point is one ansatz-and-task pair that trains above chance, with the composite audit index $\Psi_{G}$ on the horizontal axis. The left panel shows the convergence rate, defined as the fraction of the total loss reduction achieved within the first ten training steps, and the right panel shows the final classification accuracy. Within the compatible regime, the composite tracks the convergence rate closely, whereas its relationship with final accuracy is weak, indicating that a higher audit score is associated with faster learning rather than a higher performance ceiling. The number of compatible pairs is small, so the relationship is read as indicative rather than as a calibrated predictor.

A finer question is whether, among compatible pairs, a higher audit score goes with better learning. Fig 6 restricts attention to the nine pairs that train above chance and plots the composite against the two training outcomes. The composite tracks the convergence rate strongly, with a Spearman correlation of 0.89, while its correlation with final accuracy is weak at 0.26. Within the compatible set, a higher audit score is therefore associated with reaching the solution faster rather than with a better solution, consistent with the audit measuring how richly and stably an ansatz organises its trajectory rather than its ultimate fitting ceiling. The number of pairs is small, so we present this as indicative rather than as a calibrated predictor, but it shows that the audit carries information about learning behaviour beyond the binary fact of compatibility, in keeping with reading $\Psi_{G}$ as a measure of symmetry-organised complexity rather than a task-agnostic quality score [35].

### 3.7. Audit cost across system size

The audit remains inexpensive over the small state-vector sizes considered here and was also timed up to *n* = 10. A full single audit takes about 0.08 seconds at *n* = 4, 0.58 at *n* = 6, 0.94 at *n* = 8, and 2.66 at *n* = 10 on a standard CPU, with projector construction adding a few seconds at *n* = 10. The growth tracks the Hilbert-space dimension, which reaches 1024 at *n* = 10, while the number of dense-sector projectors grows only linearly, reaching 28. Because the components are evaluated via projector and gate actions on the state vector rather than via dense operator products, the method remains tractable across this range on commodity hardware, and the dense state-vector representation, rather than the audit arithmetic, sets the ceiling. Reaching the larger systems that many quantum machine learning problems target would mean replacing that representation rather than changing the audit logic.

The occupation and coherence components are expectations of projector actions on states and so admit unbiased estimation from measurement samples at a cost that does not grow with dimension, while for ansätze and inputs of bounded entanglement, a tensor-network representation would let the same actions be evaluated without forming the full state vector. The unitary-level check is more demanding, since it averages a commutator norm over parameters and group elements, but it is expressible the same way and so admits the same treatment. Lifting the dense-simulation ceiling along these lines is the main implementation step toward auditing at near-term hardware scale, while leaving the definitions unchanged.

## 4. Discussion

PsiAudit adds a target-symmetry layer to the existing diagnostics for equivariant quantum neural networks, reporting four normalised components and a configurable composite that describes how an ansatz organises its trajectory under a chosen symmetry. Our results show that this captures information that expressibility, gradient variance, and Meyer-Wallach *Q* do not, since ansätze that look alike under those measures can separate clearly in the component profile through the sectoral fluctuation and the generator-sum compliance. The framework is meant to complement the standard diagnostics rather than replace them, and it suggests an additive workflow in which a candidate ansatz is first screened with the usual measures and then audited under its intended input regime, reading the component profile rather than the scalar alone. A low $S_{G}$ signals generator-level misalignment, a high $S_{G}$ with large $M_{G}$ signals trajectory-level breaking that the proxy hides, and a high $S_{G}$ with small $H_{G}$ and $D_{G}$ signals a compliant but operationally inactive ansatz.

A central finding is that the composite depends on the input regime. An exactly equivariant ansatz gives $\Psi_{G}=0$ under sector-confined initialisation because the input never activates the relevant sectors, and recovers non-trivial values once the input spans several sectors. This is not a failure of the architecture but a reminder that ansatz design and input choice must be assessed together, in line with the broader view that inductive bias is shaped by data encoding and initialisation choices [13–16]. The high score of the $S_{n}$-equivariant ansatz should be read in the same conditional spirit. It attains the highest composite and the widest seed spread, but this reflects rich orbit occupation under its natural target in the multi-sector regime rather than general superiority, as the compatibility study makes concrete, the same ansatz solving the permutation and excitation tasks while only partially solving the collective-spin one. Audit standing and task performance are both conditional on alignment among the architecture, the target symmetry, and the problem, which is why reported audit values should always travel with the target group and regime that produced them.

The software supports these findings. PsiAudit is distributed as a Python package with a stable API and a reproducible notebook, and each run exports raw CSV outputs, sector heatmaps, figure-generation code, and a metadata record of configuration and package versions, with a unit-test suite covering the projectors, the compliance defect, and the normalisation bounds, contributing a focused audit toolkit to the wider open-source quantum-computing ecosystem [36–41].

Several limitations should be kept in view. The study is simulation-only, using exact state-vector simulation, and does not model hardware noise or noise-induced barren plateaus [11]. The behaviour of the audit under realistic device noise, where the circuit is described by a quantum channel rather than a pure unitary, is taken up directly in a related study that introduces a channel-level symmetry-breaking diagnostic for noisy equivariant circuits [31], and integrating that noise-aware reading into the present pipeline is a natural next step.

The present toolkit should accordingly be read as the noiseless state-vector member of a broader diagnostic family, while the channel-level diagnostic of the companion study provides the corresponding route for noisy implementations, so the two together span the ideal-simulation and hardware-facing regimes. The main results run to *n* = 8, where the replication and comparative diagnostics are complete, though single audits remain inexpensive at *n* = 10, and the stability we report, while established across twenty seeds and three sizes, remains a finite-size result at one depth. The implementation currently supports only *U*(1), *SU*(2), and $S_{n}$ at the Hamming-weight orbit level, and extensions to point groups, spatial symmetries, and approximate equivariance [21,42] will require additional projectors and generators. The $S_{n}$ audit should be read specifically as a Hamming-weight orbit audit, since the orbit decomposition captures the trivial-irrep occupation relevant here but does not resolve the standard (n−1,1) irrep, the sign irrep, or higher Young-diagram irreps, for which a Schur-Weyl decomposition would be needed [33]. The composite depends on the weight vector and the penalty, which is why the components are always reported alongside it. Although the sensitivity analysis shows that the induced ranking is robust to both in the present experiment, this need not hold in every setting. The within-multiplicity-space term $D_{G}^{mult}$ in Equation (4) also saturates at its clip cap for multiplicity above one and is nearly constant across ansätze for n≥6. As a result, the discriminative power of $D_{G}$ comes mainly from $D_{G}^{inter}$, and a dimension-aware normalisation that divides by $\sqrt{d_{\lambda }(d_{\lambda }-1)}$ would remove this saturation in a future version. For this reason, users scaling the audit to larger qubit counts are advised to read the inter-sector term $D_{G}^{inter}$ and the full four-component profile directly rather than relying on the combined $D_{G}$ value or the composite alone, since the saturating multiplicity-space contribution can mask differences between ansätze that the inter-sector term still resolves.

A further limitation concerns the compliance factor $S_{G}$, which the main audit computes from the generator-sum representation $H_{𝒜}$ rather than from the realised ansatz unitary, yielding a fast, parameter-independent proxy that may miss instance-level violations. The *U*(1)-broken ansatz shows the gap, since its local-X rotations violate *U*(1) at the unitary level, yet the proxy still returns $S_{G}=0.972$. A complementary unitary-level check addresses this, defined as the parameter-averaged commutator deviation,


$$\begin{array}{c} {\bar{\Delta }}_{G}^{\left(U\right)}\;=\;{𝔼}_{\theta ,g}\;\frac{\;{\left\|U(\theta )\;R(g)-R(g)\;U(\theta )\right\|}_{F}\;}{\;{\left\|U(\theta )\right\|}_{F}\;{\left\|R(g)\right\|}_{F}\;}. \end{array}$$ (8)

The toolkit computes this for the five natural-group cases. Averaged over seeds at *n* = 6 in Regime B, it is zero for the three equivariant ansätze, 0.067 for the hardware-efficient baseline, and 0.027 for the *U*(1)-broken ansatz, giving unitary-level compliances of 1.000 and 0.922, respectively, a sharper separation than the proxy provides, and being structural, it carries no seed variation. The two measures answer related but distinct questions, since $S_{G}$ probes the algebra of the gate generators while the unitary-level check probes the commutation of the realised circuit, so the package reports both and treats the unitary-level check as the more stringent.

The relationship between the audit and task performance warrants careful consideration. We do not claim that a larger $\Psi_{G}$ implies higher accuracy, lower cost, or better hardware performance on an arbitrary problem, and the compatibility study shows why such a claim would be ill-posed, since performance is relative to whether the task aligns with the architecture’s symmetry and no single task is fair across symmetry classes. What the study does show is that the audit speaks to that alignment, each architecture performing well precisely on tasks whose discriminating structure its symmetry preserves. The index is therefore best read as a pre-deployment diagnostic of symmetry organisation under a chosen target group and regime. Useful extensions include noise-aware audits, building on the channel-level diagnostics of the companion study [31], integration with established quantum software frameworks, user-defined sector projectors, and data-embedding audits that jointly assess symmetry organisation across the encoding and circuit layers [14–16].

## 5. Conclusions

PsiAudit is an open-source toolkit for auditing symmetry-organised complexity in equivariant quantum neural networks. Given an ansatz, a target group, and a state trajectory, it reports sector occupation, cross-sector coherence, sectoral fluctuation, and generator-sum compliance. These components are combined into a configurable composite score, which should be read as a practical dashboard rather than as an absolute measure of model quality.

The results show that PsiAudit captures symmetry-related behaviour that standard diagnostics can miss. An equivariant ansatz can receive a zero score when the input is confined to a single sector, but recover a non-trivial profile when the input activates several sectors. The audit therefore assesses the ansatz and the input regime together. It also separates ansätze that appear similar under expressibility, trainability, and entanglement diagnostics, mainly through sectoral fluctuation and generator-sum compliance. The added unitary-level check further clarifies cases where the generator-sum proxy is less sensitive.

Across 20 seeds and 3 system sizes, the audit profiles remain stable, with the two fully equivariant families exhibiting deterministic behaviour. The ranking is robust to changes in the component weights and compliance penalty. The compatibility study also shows that task performance for equivariant models follows the alignment between the task and the architecture’s symmetry, which is the behaviour the audit is designed to characterise.

PsiAudit is intended as a pre-deployment screening tool, not as a replacement for task-specific benchmarking. It adds a reproducible target-symmetry layer to standard expressibility, trainability, and entanglement checks, and helps relate an architecture’s symmetry organisation to the family of tasks it is likely to suit. Future work will focus on scaling the audit to larger systems using tensor-network or sparse-projector methods, extending the supported symmetries beyond *U*(1), *SU*(2), and the $S_{n}$ orbit level, implementing a full $S_{n}$-irrep decomposition, and refining the within-multiplicity-space coherence term with a dimension-aware normalisation.

## Supporting information

S1 Appendix Appendix A. Mathematical definitions and implementation details.Appendix B. Software API and example usage.
(PDF)
