# Supplementary material for: PsiAudit: An open-source toolkit for auditing symmetry-organised complexity in equivariant quantum neural networks
Source: PLoS One. 2026 Jul 17;21(7):e0353739. doi: 10.1371/journal.pone.0353739 (PMC13379041; doi:10.1371/journal.pone.0353739)
Supplement: S1 Appendix — Mathematical definitions and implementation details.Appendix B. Software API and example usage. (PDF) [file pone.0353739.s001.pdf]

Appendix A. Mathematical definitions and implementation details

This appendix gives the formal mathematical definitions of the audit primitives and the constructions of the sector projectors used by the package.

A.1 Sector projector constructions

For a system of n qubits the Hilbert space $\mathcal{H} = (\mathbb{C}^2)^{\otimes n}$ has dimension $D = 2^n$. We build sector projectors as orthogonal idempotents $\{P_\lambda\}_{\lambda \in \Lambda_G}$ that satisfy $P_\lambda^2 = P_\lambda$, $P_\lambda P_{\lambda'} = 0$ for $\lambda \neq \lambda'$, and $\sum_\lambda P_\lambda = \mathbb{1}_D$.

For $U(1)$, the projectors are diagonal in the computational basis. The Hamming-weight projector P_k acts as,

$$P_k = \sum_{x \in \{0,1\}^n,\, |x| = k} |x\rangle\langle x|, \qquad k = 0, \ldots, n, \tag{9}$$

with $|x|$ the Hamming weight of the bit-string x. Idempotence and completeness follow immediately, and the symmetry generator is the number operator $N = \frac{1}{2}\sum_{q=0}^{n-1}(I - Z_q)$ acting as $NP_k = k\,P_k$. For the product-state initialisation of Equation (7), the expected occupation in sector k is the convolution of independent Bernoulli outcomes with success probabilities $\sin^2(\alpha_q/2)$, which is straightforward to evaluate analytically. For typical trajectory-step angles near $\alpha \approx \pi/4$, the per-qubit excitation probability is approximately 0.146, which for $n = 6$ places the expected occupation peak in the $k = 1$ sector.

For $SU(2)$, the projectors are constructed from the eigenstates of the total-spin operator S^2. The operators S_x, S_y, S_z defined as $S_\alpha = \frac{1}{2}\sum_q \sigma_q^\alpha$ generate the Lie algebra $\mathfrak{su}(2)$ and satisfy $[S_\alpha, S_\beta] = i\epsilon_{\alpha\beta\gamma}S_\gamma$. We diagonalise $S^2 = S_x^2 + S_y^2 + S_z^2$ and group eigenstates by total-spin label j via the eigenvalue identity $S^2|\psi_j\rangle = j(j+1)|\psi_j\rangle$. Numerical clustering is performed by rounding j to half-integer multiples and guarding against the floating-point negative zero. The projector onto the j-sector is then $P_j = V_j V_j^\dagger$ where V_j is the matrix of eigenvectors with eigenvalue $j(j+1)$. For $n = 6$ the Schur-Weyl decomposition gives multiplicities $(m_0, m_1, m_2, m_3) = (5, 9, 5, 1)$ and irrep dimensions $(1, 3, 5, 7)$, so the sector dimensions are $(5, 27, 25, 7)$ and sum to 64. The set $\{S_x, S_y, S_z\}$ enters the compliance computation as the generators in Equation (6). The product-state initialisation of Equation (7) is not, in general, supported in a single j sector, which explains the non-zero $j = 2$ contribution visible in Figure 1.

For S_n the implementation uses the Hamming-weight orbit decomposition. The S_n action on the computational basis groups bit-strings into orbits indexed by the Hamming weight $|x| \in \{0, \ldots, n\}$, and the orbit projector is identical to the corresponding $U(1)$ Hamming-weight projector. The generators used in the compliance computation are the unitaries that implement adjacent transpositions $(i, i+1)$ for $i = 0, \ldots, n-2$. These generate the full symmetric group. As discussed in the main text, the orbit decomposition is a simplification of the full Schur-Weyl irrep decomposition and is sufficient for the audit primitives implemented here.

A.2 Generator-sum compliance defect

Given the generator set $\{G_a\}_{a=1}^{n_g}$ of the target group, and an effective ansatz Hamiltonian $H_{\mathcal{A}}$ defined as the sum of the individual gate generators that appear in the parameterised circuit, the normalised commutator defect of Equation (6) and the compliance factor $S_G = \exp(-\gamma\,\Delta_G)$ together provide a fast structural proxy. Two properties are immediate. First, $S_G \in (0,1]$, with $S_G = 1$ if and only if $H_{\mathcal{A}}$ commutes with every G_a in the generator set. Second, S_G is invariant under non-zero rescaling of $H_{\mathcal{A}}$ or any G_a, because both numerator and denominator scale identically. The construction is therefore generator-set-normalised and operator-norm-invariant. The corresponding unitary-level diagnostic of Equation (8), which the toolkit reports for the natural-group cases alongside the generator-sum compliance, replaces $H_{\mathcal{A}}$ by the parameter-dependent unitary $U(\theta)$ averaged over a parameter ensemble. The generator-sum compliance and the unitary-level compliance answer related but distinct questions, and the package can report both.

A.3 Bounds on the audit components

Each of the four components is bounded on $[0,1]$ by construction. For H_G the bound follows from Shannon entropy on a K-cell distribution, which lies on $[0,\log K]$, divided by $\log K$. For D_G^{inter}, the cross-sector Frobenius mass is bounded by $\frac{1}{2}$ for any pure state with sector probabilities summing to one, which justifies the empirical normalisation factor of two used in the implementation. The clip to $[0,1]$ guarantees the bound regardless of state-ensemble pathologies. For D_G^{mult}, the within-sector off-diagonal Frobenius mass relative to the sector trace is bounded similarly. For M_G, the inverse participation ratio R_t lies on $[1/K,1]$, so its standard deviation is bounded by half the range, which justifies the empirical normalisation factor of two. Saturation of D_G^{mult} at the empirical clip is discussed in the main text as a methodological caveat. The complementary stability quantity $1 - M_G$ used in the composite of Equation (1) inherits the same bounds.

A.4 Self-test battery

The package includes a unit test suite that runs the following correctness checks. Sector projectors are tested for idempotence with $\|P_\lambda^2 - P_\lambda\|_F < 10^{-10}$ and completeness with $\|\sum_\lambda P_\lambda - \mathbb{1}_D\|_F < 10^{-9}$. Compliance is tested against equivariant Hamiltonian benchmarks. The $U(1)$ ansatz Hamiltonian must commute with the number operator to floating-point precision, and the $SU(2)$ ansatz Hamiltonian must commute with each of S_x, S_y, S_z to the same tolerance. The broken ansatz Hamiltonian must exhibit a nonzero defect with respect to the $U(1)$ generator. Meyer-Wallach Q is calibrated on the product state with $Q = 0$ and the n-qubit GHZ state with $Q = 1$. The tests pass at all system sizes used in this paper. The validation suite additionally checks the symmetric Dicke state sector assignments for $SU(2)$, verifying that each $|W_k\rangle$ on n qubits places probability one in the $j = n/2$ sector and zero in all other j sectors, which is a direct test that the S^2 projector construction returns the expected representation-theoretic outcome on a known state family.

Appendix B. Software API and example usage

This appendix provides a brief tour of the package layout and a minimal working example showing how a user audits a custom ansatz against a chosen target group and trajectory regime.

B.1 Package layout

The package is organised into three modules that import only a common scientific stack. The core module `psiaudit_core` provides the linear-algebra primitives, the projector constructions for the three supported target groups, and the four audit components. The ansatz library `psiaudit_ansatz` provides the five reference ansätze together with their effective Hamiltonians and a registry that lets the audit pipeline iterate over them by name. The trajectory builders module `psiaudit_traj` provides the two regimes used in this paper, the random-parameter sampler used by the comparative diagnostics, and the gradient-variance helper. The reproducible Python notebook composes these modules into the experimental design described in Section 2.6 and writes the figures, tables, and metadata that constitute the empirical study.

B.2 Audit of a user-supplied ansatz

A user audits a custom ansatz by supplying a callable that returns the output state of the ansatz, given a parameter vector and an optional initial state. The signature is `apply(theta, n, L, init=None)`. The audit pipeline then uses the existing trajectory builders and projector constructions. A minimal example is given below.

```
import numpy as np
from psiaudit_core import (
    Psi_G_full, excitation_projectors,
        excitation_number_operator,
)
from psiaudit_traj import trajectory_B_multi_sector

# 1. Define a custom ansatz. The signature must accept (theta,
    n, L, init=...).
def my_ansatz(theta, n, L, init=None):
    psi = init if init is not None else _ket0(n)
    for layer in range(L):
        # apply your gates here, parameterised by theta[...]
        ...
    return psi

def my_n_params(n, L):
    return 6 * n * L   # for example

# 2. Build the projectors and the generator set for the target
    group.
n, L, T = 6, 2, 40
proj_u1, _ = excitation_projectors(n)
N = excitation_number_operator(n)
generators = [N]

# 3. Build a state trajectory in Regime B (multi-sector initial
     state).
states = trajectory_B_multi_sector(my_ansatz, n, L, my_n_params
    ,
```

```python
                                    T=T, seed=7)

# 4. Define an effective Hamiltonian for the generator-level
    compliance test.
H_eff = ...    # sum of the gate generators used by the ansatz

# 5. Compute the audit report.
report = Psi_G_full(states, proj_u1, H_eff, generators,
                    weights=(0.40, 0.35, 0.25), gamma=3.0)

print("H_G  =", report["H_G"])
print("D_G  =", report["D_G"])
print("M_G  =", report["M_G"])
print("S_G  =", report["S_G"])
print("Psi_G=", report["Psi_G"])
```

The returned dictionary also contains the underlying sector-occupation trajectory, which the user can plot as a heatmap with the helper functions provided in the package. The same pattern applies for the $SU(2)$ and S_n targets, with `su2_projectors(n)` and `sn_orbit_projectors(n)` replacing the $U(1)$ projector constructor and the corresponding generator sets returned alongside the projectors. A user wishing to audit against a custom target group can provide the projector and generator lists directly, without modifying the package.
