# Supplementary material for: PsiAudit: An open-source toolkit for auditing symmetry-organised complexity in equivariant quantum neural networks
Source: PLoS One. 2026 Jul 17;21(7):e0353739. doi: 10.1371/journal.pone.0353739 (PMC13379041; doi:10.1371/journal.pone.0353739)
Supplement: Attachment — Submitted filename: Response_to_Reviewers.pdf [file pone.0353739.s003.pdf]

Response to Reviewers

PsiAudit: An open-source toolkit for auditing symmetry-organised complexity in equivariant quantum neural networks

H. Ugail and N. Howard

Manuscript ID: PONE-D-26-26966

We thank the Editor and both reviewers for their careful and constructive assessment of our manuscript. We are pleased that both reviewers found the work technically sound, statistically appropriate, supported by available data, and clearly written, and that both recommended only minor improvements. In the revised manuscript, we have clarified the scope and naming of the toolkit, surfaced the validation of the simulator, promoted the unitary-level compliance check to a reported pipeline output, added guidance for interpreting the coherence component at larger system sizes, and strengthened the discussion of noisy-hardware extensions. All changes are marked in blue in the tracked-changes manuscript. A point-by-point response follows.

Summary of Major Revisions

The revision addresses every point raised by both reviewers together with the journal editorial requirements. The most important changes are summarised below and are then treated individually in the per-reviewer responses.

1. Naming and scope of the toolkit clarified

In response to Reviewer 1, we added a sentence at the first mention of PsiAudit in the Introduction that explains the name, stating that the prefix refers to the quantum state vector whose symmetry organisation is measured and that the term audit denotes a pre-deployment diagnostic screen of ansatz behaviour rather than a trained-model evaluation. We have retained the name to preserve continuity with the released software and related publications.

2. Validation of the simulator made explicit

In response to Reviewer 1, we made the validation of the audit primitives explicit in the experimental-design subsection. The revised text states that no reported audit quantity rests on a single realisation, since every quantity is replicated across twenty independent seeds with standard deviations and bootstrap confidence intervals, and it describes the unit-test battery that verifies the projector constructions, the compliance defect, the entanglement calibration, and the normalisation bounds at every system size used.

3. Unitary-level compliance check promoted to a reported output

In response to Reviewer 2, the parameter-averaged commutator deviation has been promoted from an optional validation step to a reported part of the audit output. The Methods now

describe it as a standard, more stringent compliance diagnostic that the toolkit reports for the natural-group cases alongside the generator-sum compliance.

4. Guidance added for the coherence component at scale

In response to Reviewer 2, we added explicit guidance on interpreting the cross-sector coherence component when scaling to larger qubit counts, advising users to read the inter-sector term and the full four-component profile directly rather than the combined value or the composite alone, and we retained the identification of a dimension-aware normalisation as the next implementation step.

5. Noisy-hardware extension strengthened

In response to Reviewer 2, we expanded the treatment of hardware noise. The revised Discussion states that the present toolkit is the noiseless state-vector member of a broader diagnostic family and cites a related study, now published in IEEE Access, that develops a channel-level symmetry-breaking diagnostic for noisy equivariant circuits, identifying its integration as a natural next step.

6. Additional corrections

We corrected a typographical error and a sentence fragment in the Discussion, both noted during our own proofreading, and we added brief modesty qualifiers to the abstract, the downstream study, and the cost section so that the added analyses are not read as stronger claims than the data support.

Response to Journal Requirements

Requirement 1. PLOS ONE style and file naming

Our response. The manuscript has been checked against the PLOS ONE formatting templates and file-naming conventions, and the revised files are named according to the journal requirements as Manuscript, Revised Manuscript with Track Changes, and Response to Reviewers.

Requirement 2. PLOS LaTeX template

Our response. The manuscript uses the PLOS ONE LaTeX template with the required formatting and bibliography style.

Requirement 3. Reviewer-suggested citations

No specific previously published works were required by the editor. Reviewer 2 noted the need to address hardware noise, which we have done by citing our companion study on channel-level diagnostics for noisy equivariant circuits, now published in IEEE Access, where it is directly relevant.

→ *Methods (Section 2.3), Discussion (limitations and future work).*

Response to Reviewer 1

We thank Reviewer 1 for the generous assessment of the manuscript and for highlighting the importance of clearly defining the scope and validation of the tool. We address each point below.

“Well written, presented and supported. It was a pleasure to read. This reviewer did not find anything wrong with the paper, and would hope only that the authors take extra care in advising the reader on the scope and limitations of the simulator.”

We are grateful for this assessment and have addressed the reviewer’s specific points below.

Naming of the toolkit

“PsiAudit as a name for the toolkit is confusing and collides with tradition for naming of tools among various disciplines from engineering to psychology. From the name of the toolkit, the reviewer would not guess its purpose, and it seems misnamed for those who have other Psi or Psy prefixed tools already in use, or audit tools.”

Our response. We thank the reviewer for raising this, and we considered the naming collision carefully. We have added a sentence at the first use of PsiAudit in the Introduction that clarifies the intended meaning, namely that the prefix refers to the quantum state vector whose symmetry organisation is being measured, in keeping with the standard physics use of the symbol, and that the term audit denotes a pre-deployment diagnostic screen of ansatz behaviour rather than a trained-model evaluation. After careful consideration we have retained the name itself, because the toolkit, its public repository, and our related papers are already released and referenced under this name, so a rename at this stage would break continuity across the published record. The added clarification removes the ambiguity at the point where a reader first meets the name.

→ *Introduction (first mention of PsiAudit).*

Scope and validation of the simulator

“Assuming that an efficient path is to simulate a structure or design before going further, and as PsiAudit is a simulator, it then conditions later exploration. Imprecision in a simulator will influence choices about what to explore. This reviewer can not evaluate the precision of the PsiAudit simulator. Researchers need to read the details on how this PsiAudit is validated.”

Our response. We agree that the precision of a screening tool matters because it conditions later exploration, and we have made the validation of the toolkit explicit and easy to find. The experimental-design subsection now states directly that no reported audit quantity rests on a single realisation, since every quantity is replicated across twenty independent random seeds and reported with standard deviations and bootstrap confidence intervals. We have also surfaced the unit-test battery in the same place, describing what it checks, namely the

idempotence and completeness of the sector projectors, the vanishing of the compliance defect for the equivariant constructions against their target generators, the calibration of the entanglement diagnostic on reference states, and the normalisation bounds of the components, and we state that these tests pass at every system size used in the study. The full validation suite is included in the public repository so that the precision can be inspected directly.

→ *Methods (experimental-design subsection); Appendix A (unit tests); public repository.*

Response to Reviewer 2

We thank Reviewer 2 for the thorough and constructive review and for the detailed summary of the toolkit's contributions. We address each of the five points in turn.

Comment 1. Generator-sum blind spot

"The proxy-based generator-sum calculation demonstrates a blind spot, yielding an overly high compliance score for circuits with late-stage local symmetry-breaking gates."

Our response. We agree, and this is precisely the behaviour the manuscript identifies and addresses. The Methods now describe the compliance factor explicitly as a fast generator-sum proxy that can look symmetric even when individual circuit instances do not commute with the target action, for example under fixed non-infinitesimal local rotations that cancel under the linear sum but not under the unitary product. The U(1)-broken reference ansatz is included precisely to expose this case, and the Results show that the generator-sum proxy returns a high compliance of 0.972 for it, while the complementary unitary-level diagnostic detects the local-X breaking clearly. The two readings are reported side by side.

→ *Methods (Section 2.3); Results (Section 3.2); Discussion.*

Comment 2. Promote the unitary-level commutator deviation

"The parameter-averaged commutator deviation check successfully flags subtle symmetry-breaking defects. Authors can consider promoting this metric from an optional validation step in the discussion to a primary, configurable choice within the core pipeline."

Our response. We have made this change. The unitary-level commutator deviation is now described in the Methods as a standard part of the audit output that the toolkit reports for the natural-group cases alongside the generator-sum compliance, and the matching description in the appendix has been updated to agree. It is presented as the more stringent of the two compliance measures, because it samples realised circuit unitaries and tests commutation of the implemented circuit directly rather than relying on the generator sum. The Discussion reports its values, namely zero for the three equivariant ansaetze and a non-zero deviation for the hardware-efficient and U(1)-broken constructions, giving a sharper separation than the proxy alone.

→ *Methods (Section 2.3); Discussion; Appendix A.2.*

Comment 3. Coherence saturation at larger qubit counts

"The internal cross-sector coherence factor hits artificial numerical saturation boundaries at larger qubit counts. Authors must clarify how users should interpret or adjust the weights of the coherence indices when scaling up circuits."

Our response. We have clarified this in the Discussion. The revised text explains that the within-multiplicity-space term of the coherence component saturates at its clip cap for multiplicity above one and becomes nearly constant across ansaetze for six or more qubits, so the discriminative power of the coherence component comes mainly from its inter-sector term. We now advise users scaling the audit to larger qubit counts to read the inter-sector term and the full four-component profile directly rather than relying on the combined coherence value or the composite alone, since the saturating multiplicity-space contribution can mask differences that the inter-sector term still resolves. We retain the identification of a dimension-aware normalisation as the next implementation step.

→ *Discussion (limitations paragraph).*

Comment 4. Schur-Weyl decomposition for higher irreducible representations

"The current permutation audit maps basic Hamming-weight orbits but cannot isolate individual multiplicity spaces for higher-order irreducible representations. Authors must outline a developmental path for incorporating a true Schur-Weyl decomposition engine to resolve complex representation structures."

Our response. We agree and have made the scope and the developmental path explicit. The manuscript consistently describes the permutation audit as an S_n Hamming-weight orbit audit, and the Discussion states that the orbit decomposition captures the trivial-irrep occupation relevant here but does not resolve the standard irrep, the sign irrep, or higher Young-diagram irreps, for which a Schur-Weyl decomposition would be required. We identify this Schur-Weyl based refinement as a defined next step for the implementation.

→ *Methods (Section 2.4); Discussion (limitations paragraph); Conclusions.*

Comment 5. Hardware noise

"The toolkit relies entirely on ideal state-vector simulations. Authors must address how environmental decoherence, readout noise, or sampling error on real near-term quantum hardware affect the baseline values of the composite score."

Our response. We agree that hardware noise is important and have expanded the limitations accordingly. The revised Discussion states that the present paper provides a noiseless state-vector audit and that realistic device noise, where the circuit is described by a quantum channel rather than a pure unitary, requires a channel-level treatment. We now cite a related study, published in IEEE Access, that develops exactly such a channel-level symmetry-breaking

diagnostic for noisy equivariant circuits, and we frame the present toolkit as the noiseless state-vector member of a broader diagnostic family whose channel-level counterpart provides the route for noisy implementations. Integration of that noise-aware reading into the present pipeline is identified as a natural next step.

→ *Methods (Section 2.3); Discussion (limitations and future work).*

We are grateful to the Editor and both reviewers for the care they have taken with the manuscript. We believe the revised version is clearer in scope, more explicit in its validation, and stronger in its treatment of compliance and noise, and we hope it is now suitable for publication in PLOS ONE.
