# Supplementary material for: PsiAudit: An open-source toolkit for auditing symmetry-organised complexity in equivariant quantum neural networks
Source: PLoS One. 2026 Jul 17;21(7):e0353739. doi: 10.1371/journal.pone.0353739 (PMC13379041; doi:10.1371/journal.pone.0353739)
Supplement: Peer review history

# Peer review: Decision Letter 0

**DOI:** 10.1371/journal.pone.0353739.r001

**Authors:** Zeheng Wang

22 Jun 2026

PONE-D-26-26966

PsiAudit: An open-source toolkit for auditing symmetry-organised complexity in equivariant quantum neural networks

PLOS One

Dear Dr. Ugail,

Thank you for submitting your manuscript to PLOS ONE. After careful consideration, we feel that it has merit but does not fully meet PLOS ONE’s publication criteria as it currently stands. Therefore, we invite you to submit a revised version of the manuscript that addresses the points raised during the review process.

Please submit your revised manuscript by Aug 21 2026 11:59PM. If you will need more time than this to complete your revisions, please reply to this message or contact the journal office at plosone@plos.org. When you're ready to submit your revision, log on to https://www.editorialmanager.com/pone/ and select the 'Submissions Needing Revision' folder to locate your manuscript file.

Please include the following items when submitting your revised manuscript:

- A letter that responds to each point raised by the academic editor and reviewer(s). You should upload this letter as a separate file labeled 'Response to Reviewers'.
- A marked-up copy of your manuscript that highlights changes made to the original version. You should upload this as a separate file labeled 'Revised Manuscript with Track Changes'.
- An unmarked version of your revised paper without tracked changes. You should upload this as a separate file labeled 'Manuscript'.

If you would like to make changes to your financial disclosure, please include your updated statement in your cover letter. Guidelines for resubmitting your figure files are available below the reviewer comments at the end of this letter.

If applicable, we recommend that you deposit your laboratory protocols in protocols.io to enhance the reproducibility of your results. Protocols.io assigns your protocol its own identifier (DOI) so that it can be cited independently in the future. For instructions see: https://journals.plos.org/plosone/s/submission-guidelines#loc-laboratory-protocols. Additionally, PLOS ONE offers an option for publishing peer-reviewed Lab Protocol articles, which describe protocols hosted on protocols.io. Read more information on sharing protocols at https://plos.org/protocols?utm_medium=editorial-email&utm_source=authorletters&utm_campaign=protocols.

As the corresponding author, your ORCID iD is verified in the submission system and will appear in the published article. PLOS supports the use of ORCID, and we encourage all coauthors to register for an ORCID iD and use it as well. Please encourage your coauthors to verify their ORCID iD within the submission system before final acceptance, as unverified ORCID iDs will not appear in the published article. *Only*  the individual author can complete the verification step; PLOS staff *cannot*  verify ORCID iDs on behalf of authors.

We look forward to receiving your revised manuscript.

Kind regards,

Zeheng Wang

Academic Editor

PLOS One

Journal Requirements:

When submitting your revision, we need you to address these additional requirements.

1. Please ensure that your manuscript meets PLOS ONE's style requirements, including those for file naming. The PLOS ONE style templates can be found at

https://journals.plos.org/plosone/s/file?id=wjVg/PLOSOne_formatting_sample_main_body.pdf and

https://journals.plos.org/plosone/s/file?id=ba62/PLOSOne_formatting_sample_title_authors_affiliations.pdf

2. Please update your submission to use the PLOS LaTeX template. The template and more information on our requirements for LaTeX submissions can be found at http://journals.plos.org/plosone/s/latex.

3. If the reviewer comments include a recommendation to cite specific previously published works, please review and evaluate these publications to determine whether they are relevant and should be cited. There is no requirement to cite these works unless the editor has indicated otherwise.

[Note: HTML markup is below. Please do not edit.]

Reviewers' comments:

Reviewer's Responses to Questions

**Comments to the Author**

1. Is the manuscript technically sound, and do the data support the conclusions?

The manuscript must describe a technically sound piece of scientific research with data that supports the conclusions. Experiments must have been conducted rigorously, with appropriate controls, replication, and sample sizes. The conclusions must be drawn appropriately based on the data presented.

Reviewer #1: Yes

Reviewer #2: Yes

**********

2. Has the statistical analysis been performed appropriately and rigorously?

Reviewer #1: Yes

Reviewer #2: Yes

**********

3. Have the authors made all data underlying the findings in their manuscript fully available?

The PLOS Data policy requires authors to make all data underlying the findings described in their manuscript fully available without restriction, with rare exception (please refer to the Data Availability Statement in the manuscript PDF file). The data should be provided as part of the manuscript or its supporting information, or deposited to a public repository. For example, in addition to summary statistics, the data points behind means, medians and variance measures should be available. If there are restrictions on publicly sharing data—e.g. participant privacy or use of data from a third party—those must be specified.

Reviewer #1: Yes

Reviewer #2: Yes

**********

4. Is the manuscript presented in an intelligible fashion and written in standard English?

PLOS ONE does not copyedit accepted manuscripts, so the language in submitted articles must be clear, correct, and unambiguous. Any typographical or grammatical errors should be corrected at revision, so please note any specific errors here.

Reviewer #1: Yes

Reviewer #2: Yes

**********

5. Review Comments to the Author

Please use the space provided to explain your answers to the questions above. You may also include additional comments for the author, including concerns about dual publication, research ethics, or publication ethics. (Please upload your review as an attachment if it exceeds 20,000 characters)

Reviewer #1: Well written, presented and supported. It was a pleasure to read; this reviewer appreciates the care and effort of the authors to develop the project and manuscript.

The scope and impact of the paper are difficult to predict in its current state.

A clear description of the software is helpful and can serve as a useful guide for researchers following in the authors' path.

Explaining symmetry 'organized' complexity is important and should be examined.

There is much to test and report in this area.

Automation is needed if we wish to explore the domain. It so follows that the authors' work reported in this paper is useful in the construction effort for tools to this purpose.

'PsiAudit' as a name for the toolkit is confusing and collides with tradition for naming of tools among various disciplines from engineering to psychology. A great scope of work in psychology, neuroscience, accounting, and social sciences exists, in which names for methods and tools collide with the name that has been chosen here.

For something as important as examining symmetry a better name can be be found.

This work needs to be viewed as a possible gatekeeper for exploration of circuits.

41 > ""The choice of ansatz affects trainability, resource requirements, inductive bias, and the range of states the model can reach [5, 6, 7].

Regardless of the tool aspect, the concept that -

619 > ""Our results show this captures information that expressibility, gradient variance, and Meyer-Wallach Q do not, since ansätze that look alike under those measures can separate clearly in the component profile

- this is valuable in the presentation of a direction for exploration.

Assuming that an efficient path is to simulate a structure or design before going further, and as PsiAudit is a simulator, it then conditions later exploration. Imprecision in a simulator will influence choices about what to explore.

This reviewer can not evaluate the precision of the PsiAudit simulator.

As a tool it is a valuable contribution.

Researchers need to read the details on how this PsiAudit is validated.

ie.

965 > .... The tests pass at all system sizes used in this paper.

Recommendations -

This reviewer did not find anything wrong with the paper, and would hope only that the authors take extra care in advising the reader on the scope and limitations of the simulator.

From the name of the toolkit, the reviewer would not guess its purpose, and it seems misnamed for those who have other 'Psi/Psy ... ' prefixed tools already in use, or 'audit' tools. This reviewer encounters both and assumes Psi/Psy to be psychology/neuroscience and Audit to be psychology/engineering/accounting.

Reviewer #2: The authors have introduced an open-source Python diagnostic toolkit, PsiAudit designed to evaluate symmetry-organised complexity within Equivariant Quantum Neural Networks. This toolkit addresses a critical diagnostic blind spot in Quantum Machine Learning (QML) by introducing four distinct, normalized structural axes: sector occupation entropy, cross-sector coherence, sectoral fluctuation, and generator-sum compliance.

The framework successfully breaks diagnostic degeneracy by distinguishing between equivariant and symmetry-broken architectures in scenarios where standard metrics yield overlapping results.

The core primitives are rigorously bounded within a zero-to-one interval using appropriate statistical normalization techniques, ensuring cross-architecture comparability.

The architecture functions out-of-the-box across highly diverse structural group paradigms, including Abelian, non-Abelian Lie, and finite-discrete groups.

Computing metrics via operator and projector actions on pure state vectors rather than forming dense density matrices keeping execution efficient and highly scalable on standard CPU hardware.

The inclusion of a public repository containing raw data, parameters, configuration seeds, and an executable Jupyter Notebook fully satisfies open-science standards.

However, there is still distinct room for improvement, and the following points should be addressed:

* The proxy-based generator-sum calculation demonstrates a blind spot, yielding an overly high compliance score for circuits with late-stage local symmetry-breaking gates.

* The parameter-averaged commutator deviation check successfully flags subtle symmetry-breaking defects. Authors can consider promoting this metric from an optional validation step in the discussion to a primary, configurable choice within the core pipeline.

* The internal cross-sector coherence factor hits artificial numerical saturation boundaries at larger qubit counts. Authors must clarify how users should interpret or adjust the weights of the coherence indices when scaling up circuits.

* The current permutation audit maps basic Hamming-weight orbits but cannot isolate individual multiplicity spaces for higher-order irreducible representations. Authors must outline a developmental path for incorporating a true Schur-Weyl decomposition engine to resolve complex representation structures.

* The toolkit relies entirely on ideal state-vector simulations. Authors must address how environmental decoherence, readout noise, or sampling error on real near-term quantum hardware affect the baseline values of the composite score.

**********

6. PLOS authors have the option to publish the peer review history of their article (what does this mean?). If published, this will include your full peer review and any attached files.

If you choose “no”, your identity will remain anonymous but your review may still be made public.

**Do you want your identity to be public for this peer review?**  For information about this choice, including consent withdrawal, please see our Privacy Policy.

Reviewer #1: No

Reviewer #2: No

**********

[NOTE: If reviewer comments were submitted as an attachment file, they will be attached to this email and accessible via the submission site. Please log into your account, locate the manuscript record, and check for the action link "View Attachments". If this link does not appear, there are no attachment files.]

To ensure your figures meet our technical requirements, please review our figure guidelines: https://journals.plos.org/plosone/s/figures

You may also use PLOS’s free figure tool, NAAS, to help you prepare publication quality figures: https://journals.plos.org/plosone/s/figures#loc-tools-for-figure-preparation.

NAAS will assess whether your figures meet our technical requirements by comparing each figure against our figure specifications.

# Author comment: Author response to Decision Letter 1

**DOI:** 10.1371/journal.pone.0353739.r002

27 Jun 2026

See attached Response to Reviewers document.

Attachment Submitted filename: Response_to_Reviewers.pdf

# Editor report: Decision Letter 1

**DOI:** 10.1371/journal.pone.0353739.r003

**Authors:** Zeheng Wang, Zeheng Wang

29 Jun 2026

PsiAudit: An open-source toolkit for auditing symmetry-organised complexity in equivariant quantum neural networks

PONE-D-26-26966R1

Dear Dr. Ugail,

We’re pleased to inform you that your manuscript has been judged scientifically suitable for publication and will be formally accepted for publication once it meets all outstanding technical requirements.

Within one week, you’ll receive an e-mail detailing the required amendments. When these have been addressed, you’ll receive a formal acceptance letter and your manuscript will be scheduled for publication.

An invoice will be generated when your article is formally accepted. Please note, if your institution has a publishing partnership with PLOS and your article meets the relevant criteria, all or part of your publication costs will be covered. Please make sure your user information is up-to-date by logging into Editorial Manager at Editorial Manager® and clicking the ‘Update My Information' link at the top of the page. For questions related to billing, please contact billing support.

If your institution or institutions have a press office, please notify them about your upcoming paper to help maximize its impact. If they’ll be preparing press materials, please inform our press team as soon as possible -- no later than 48 hours after receiving the formal acceptance. Your manuscript will remain under strict press embargo until 2 pm Eastern Time on the date of publication. For more information, please contact onepress@plos.org.

Kind regards,

Zeheng Wang

Academic Editor

PLOS One

Additional Editor Comments (optional):

Reviewers' comments:

# Editor report: Acceptance letter

**DOI:** 10.1371/journal.pone.0353739.r004

**Authors:** Zeheng Wang, Zeheng Wang

PONE-D-26-26966R1

PLOS One

Dear Dr. Ugail,

I'm pleased to inform you that your manuscript has been deemed suitable for publication in PLOS One. Congratulations! Your manuscript is now being handed over to our production team.

At this stage, our production department will prepare your paper for publication. This includes ensuring the following:

* All references, tables, and figures are properly cited

* All relevant supporting information is included in the manuscript submission,

* There are no issues that prevent the paper from being properly typeset

You will receive further instructions from the production team, including instructions on how to review your proof when it is ready. Please keep in mind that we are working through a large volume of accepted articles, so please give us a few days to review your paper and let you know the next and final steps.

Lastly, if your institution or institutions have a press office, please let them know about your upcoming paper now to help maximize its impact. If they'll be preparing press materials, please inform our press team within the next 48 hours. Your manuscript will remain under strict press embargo until 2 pm Eastern Time on the date of publication. For more information, please contact onepress@plos.org.

You will receive an invoice from PLOS for your publication fee after your manuscript has reached the completed accept phase. If you receive an email requesting payment before acceptance or for any other service, this may be a phishing scheme. Learn how to identify phishing emails and protect your accounts at https://explore.plos.org/phishing.

If we can help with anything else, please email us at customercare@plos.org.

Thank you for submitting your work to PLOS ONE and supporting open access.

Kind regards,

PLOS ONE Editorial Office Staff

on behalf of

Dr. Zeheng Wang

Academic Editor

PLOS One
